# Molecular Characterization and Evolution Analysis of Two Forms of TLR5 and TLR13 Genes Base on *Larimichthys crocea* Genome Data

**DOI:** 10.1155/2020/4895037

**Published:** 2020-12-09

**Authors:** Lihua Jiang, Liyi Pei, Ping Wang, Liqin Liu, Gong Li, Binjian Liu, Zhenming Lǚ, Tabata Hiromasa, Hao Pan, Atsushi Ogura

**Affiliations:** ^1^National Engineering Research Center for Facilitated Marine Aquaculture, Zhejiang Ocean University, No. 1, South Haida Road, Dinghai District, Zhoushan, China; ^2^Nagahama Institute of Bioscience and Technology, Tamura, Nagahama, Shiga, Japan

## Abstract

TLRs (Toll-like receptors) are essential in host defense against pathogens. There are two types of TLR5, namely, membrane form of TLR5 (TLR5M) and soluble form of TLR5 (TLR5S), both of which perform a crucial role in flagellin response. TLR13 is a TLR that localizes to endosomes and recognizes nucleic acids released by internal microorganisms, including viruses, bacteria, and fungi. Here, the full-length coding sequence (CDS), protein structure, and immune response and subcellular localization of TLR5 (TLR5S) and TLR13 were characterized in large yellow croaker (*Larimichthys crocea*). These TLRs share high sequence homology with other ichthyic TLRs, while also having their own characters; qtPCR was determined and the results found that the three genes were constitutively expressed in all examined tissues: TLR5M was highly expressed in the spleen and liver; TLR13 expression was high in the kidney, liver, and spleen. And TLRs were upregulated following stimulation with *Vibrio parahaemolyticus* in the liver, spleen, and kidney. Immunofluorescence staining revealed that TLR5M were localized in the cytoplasm, while TLR5S and TLR13 were in the endosome. The evolutionary analysis has shown that TLR13 was clustered with TLR11, 19, 20, 21, and 22, while TLR5 and TLR3 were classified into a group; these results suggest that TLRs are vital in the defense of *L. crocea* against bacterial infection and further increase our understanding of TLR function in innate immunity in teleosts.

## 1. Introduction

TLRs (Toll-like receptors) were first discovered in Drosophila embryos and control the formation of the dorsal-ventral polarity of early embryos [1]. Further studies have shown that TLRs are involved in the synthesis of antimicrobial peptides and are capable of antifungal infections [2]. In 1997, the Toll ortholog of Drosophila melanogaster was identified in humans and proved to be essential for the activation of the innate or adaptive immunity [3, 4].

TLRs are type I transmembrane proteins composed of extracellular, transmembrane, and intracellular signal domains: the extracellular domain has a repetitive leucine-rich repeat (LRR); the ligand-induced dimerization of TLR triggers the recruitment of adaptor proteins to the intracellular Toll/IL-1 receptor homology domain (TIR), thereby initiating signal transduction [5]. The extracellular domain of the TLR protein usually contains 16-28 LRRs, which are responsible for binding PAMPs and involve a variety of physiological functions including immune response, signal transduction, cell cycle regulation, and enzyme regulation [6]. A single LRR module typically has 20-30 amino acids in length and consists of a variable portion and a conserved “LxxLxLxxN” motif that allows the extracellular domain to form a special horseshoe [5, 7, 8]. The LRRNT and LRRCT modules in the N-terminus and C-terminus of the LRR domain do not function as LRR, but their cysteines are aggregated to form disulfide bonds and protect the hydrophobic core of the protein from exposure to the solvent, stabilizing the protein structure [7].

The first fish TLR gene was discovered in goldfish in 2002, and several TLRs were retrieved from the genomic data of pufferfish and zebrafish, respectively, in 2004 [9]. So far, more than 20 TLRs have been identified from more than ten species of teleost fish, namely, TLR1, 2, 3, 4, 5M, 5S, 7, 8, 9, 13, 14, 18, 19, 20, 21, 22, 23, 24, 25, 26, and 27 [10, 11]. Generally, there are no TLR6 and TLR10 in fish, while TLR14 and TLR22 are only found in fish [12]. TLR1-13, which are mutual between humans and fish, may have different ligands and functions in fish and mammals. For example, human TLR3 can only recognize viruses, while fish TLR3 can not only respond to virus invasion but also recognize bacterial PAMPs; fish TLR4 may be an inhibitor of the MYD88-dependent signaling pathway [13, 14]. Human TLRs can be broadly divided into two major subgroups: TLRs 1, 2, 4, 5, 6, and 10 located on the cell surface identify lipoproteins, lipopolysaccharides, or flagellin on the surface of microorganisms, while TLR3, 7, 8, and 9 locate on the endosomes or lysosomes and recognize the nucleic acid sequence of the microorganism [15]. In addition to the cross-model (TLR5M), fish TLR5 also has soluble TLR5 (TLR5S), both of which recognize flagellin on the surface of pathogenic bacteria [16].

TLR5-ligand interaction in mammals, TLR5, is primarily responsible for the detection of a kind of flagellin in bacterial flagella and the specific identification of constant functional domains that are relatively conserved between different bacteria [17]. To fish, they have two forms of TLR5, TLR5M (membrane form) and TLR5S (soluble form), which are responsible for identifying bacterial flagellin [18]. Fish TLR5M is homologous to mammalian TLR5 and contains the LRR domain, transmembrane region, and TIR domain typical of TLR. However, TLR5 is unique to fish and lacks transmembrane and TIR regions. Studies have shown that the recombinant TLR5S of *Sparus aurata* can bind to flagellin of *Vibrio anguillarum*, and then, TLR5M activates the inflammatory response. But it is still controversial whether the exact function of TLR5S is to enhance the innate immune response or to avoid the excessive inflammatory response of TLR5M [19]. Therefore, the molecular mechanism of fish TLR5 in the regulation of flagellar-mediated immune responses requires further investigation.

As per previous studies, TLR13 is a receptor for bacterial RNA; small-interfering RNA against TLR13 reduced cytokine induction by bRNA in DCs [20]; additionally, TLR13-ligand interaction TLR3, TLR7-9, TLR11, and TLR13 are TLRs localized to endosomes, which basically recognize nucleic acids released by intracellular microorganisms, including viruses, bacteria, and fungi. It has been demonstrated that mouse TLR13 can recognize the highly conserved 5′-CGGAAAGACC-3′ motif of bacterial 23S rRNA [6, 21]. The ssRAN oligomer mutation assay demonstrated that most of the nucleotides in the conserved sequence of the 23S rRNA of bacteria, especially the last eight nucleotides, are essential for triggering TLR13-mediated signaling [21]. In addition, vesicular stomatitis virus (VSV) has also been shown to induce a TLR13-mediated immune response [22]. However, the chemical properties that lead to the activation of TLR13 have been difficult to determine.

*Larimichthys crocea* is one of the most economically important fish endemic in the eastern and southern coastal waters of China. It has a well-developed innate immune system [23]. However, the wild stocks of *L. crocea* are seriously damaged by overfishing, while the cultured *L. crocea* is vulnerable to various pathogens [24, 25]. At present, there are more than 20 kinds of TLRs found in fish, and only 6 *L. crocea* TLRs are published, while TLR5, which has two forms, namely, membrane form (TLR5M) and soluble form (TLR5S), was poorly understood; meantime, TLR13, one of the important receptor, and the mechanism of innate immunity of large yellow croakers need further study. Therefore, this study was focused on three genes, and the results will contribute to our understanding of the immune system in fish.

## 2. Materials and Methods

### 2.1. Fish and Tissue Sampling

The examined fishes (weight 800 ± 15 g), obtained from Zhejiang Dahaiyang Technology Co. Ltd. (Zhoushan, Zhejiang Province, China), were clinically healthy. They were maintained at 25°C in an aerated seawater tank and fed a commercial diet for two weeks prior to the start of the experiments. The water in the tank was changed daily.

For the basal tissue expression analysis, tissues (muscle, spleen, liver, kidney, brain, heart, intestine, gill, swimming bladder, skin, fin, and eye) were isolated from six unchallenged *L. crocea* after being anaesthetized by immersion in MS222.

To investigate the transcriptional modulations of lcTLRs, two independent groups of 100 individuals were infected with *V. parahaemolyticus* (1 × 10^8^ CFU/mL, resuspended in PBS, pH 7.4) and PBS (as control) at a dose of 300 *μ*L/200 g, respectively. Then, the liver, kidney, and spleen tissues were collected from three fishes per group, at 0 h, 6 h, 12 h, 24 h, 36 h, 48 h, and 72 h postinjection. All procedures were performed under the guidelines of the Regulations for the Administration of Laboratory Animals (Decree No. 2 of the State Science and Technology Commission of the People's Republic of China, November 14, 1988) and were approved by the Animal Ethics Committee of Zhejiang Ocean University (Zhoushan, China).

### 2.2. RNA Isolation and Molecular Cloning

Total RNA was isolated from examined tissues using the Trizol Total RNA Kit (Invitrogen, USA) following the manufacturer's instructions. Then, the concentration was measured by a UV-spectrophotometer (Eppendorf, Germany), and the quality of RNA was analyzed by 1% agarose gel by visualizing the intensity of 18 and 28s ribosomal RNA. After that, cDNA was synthesized using the M-MLV RTase cDNA Synthesis Kit (TaKaRa, Japan) following the guidelines of its manufacturer's instructions. Finally, using the cDNAs as templates, PCR amplification was conducted using a thermal cycler (Bio-Rad, USA) under the following conditions: 50°C for 2 min, 95°C for 30 s, and then 40 cycles of 95°C for 15 s, 58°C for 45 s, and 95°C for 15 s, followed by a final extension at 72°C for 5 min.

### 2.3. Quantitative Real-Time PCR (qRT-PCR) and Statistical Analysis

Tissue-specific distribution and temporal mRNA expression of TLR5S, TLR5M, and TLR13 upon immune challenge were determined by qRT-PCR. A sample of 2 g of total RNA was reverse transcribed in a final volume of 20 *μ*L using the PrimeScriptTM RT reagent kit (Tli RNaseH Plus, TaKaRa, China) following the manufacturer's instructions. Then, qRT-PCR was carried out in a reaction mixture of 20 *μ*L, containing 0.8 *μ*L primer-F (10 *μ*mol/L), 0.8 *μ*L primer-R (10 *μ*mol/L), 8 *μ*L 2x SYBR ®Premix Ex TaqTM II, 1 *μ*L cDNA sample (100 ng/*μ*L), 0.4 *μ*L ROX II, and 9 *μ*L ddH_2_O (the reagent concentration refers to its manufacturer's instructions) in triplicate. The reference gene *β*-actin was used to normalize the expressions of TLR5M, TLR5S, and TLR13. Primers for qRT-PCR (Table 1) were designed using the Primer5.064 software using the CDS of lcTLRs and *β*-actin predicted by the GeneScan online tool.

All data were analyzed using the 2^-*ΔΔ*CT^ method (Livak and Schmittgen, 2001) to obtain the relative mRNA expression. The mRNA expression in muscle was treated as a control for comparisons with various tissues, and the 0 h time point comprised the control for the expressions post *V. parahaemolyticus* infection. Two-tailed Student's *t*-test was performed to determine the statistical significance of differences observed between the experimental and control groups using SPSS Statistics 19 (IBM). ^∗^*p* < 0.05 and ^∗∗^*p* < 0.01 were considered statistically significant and indicated in Results.

### 2.4. Protein Localization

We used pcDNA3.1-C-eGFP plasmids (Genscript, Nanjing, China) to transform the overexpressed protein TLR5S, TLR5M, and TLR13 with green fluorescence. Transfection was using FTX and PLUS reagents (InvitrogenTM, Calif., USA). Cells were seeded in 24-well plates and cultured. DAPI was used to locate the nucleus, and Dil was used to locate the cytomembrane. The localization of protein TLR5S, TLR5M, and TLR13 was observed under a Nikon TE2000 microscope.

### 2.5. Sequence Analysis

The gene sequences of TLR5M, TLR5S, and TLR13 were obtained from the *L. crocea* whole-genome data [24]. Their coding sequences and the amino acid sequences were predicted using GeneScan (http://genes.mit.edu/GENSCAN.html) and ORFfinder (https://www.ncbi.nlm.nih.gov/orffinder/). The theoretical molecular weight (MW) and isoelectric point (pI) were determined by Expasy-ProtParam (http://web.expasy.org/protparam/). The domains of the proteins were predicted using the online tool SMART (http://smart.embl-heidelberg.de) and pictured by IBS (http://ibs.biocuckoo.org). The secondary structure and tertiary structure were predicted using Phyre2 (http://www.sbg.bio.ic.ac.uk/~phyre2/html/page.cgi?id=index) [26]. The homology genes were determined with BLASTp (https://blast.ncbi.nlm.nih.gov/Blast.cgi?PAGE=Proteins) and in the Gene Database (https://www.ncbi.nlm.nih.gov/gene/). Multiple sequence alignments were performed with MEGA7 using ClustalW and ESPript3 (http://espript.ibcp.fr/ESPript/ESPript/index.php). The phylogenetic tree was constructed by MEGA7, using the neighbor-joining method.

## 3. Results

### 3.1. Sequence Characteristics and Protein Structure Prediction

The coding region (CDS) of the TLR5M gene was deduced to be 2658 bp in length and encodes a protein of 885 aa with a predicted MW of 101.369 kDa and a pI of 5.69.

**Figure 1:**
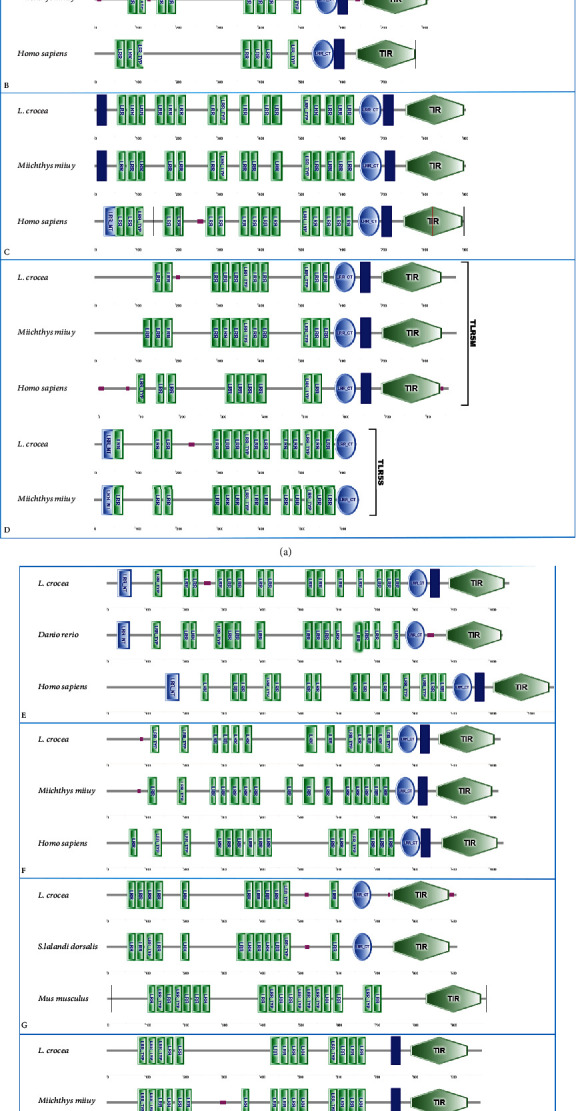


The 1926 bp length CDS of the TLR5S gene encoded a protein of 641 aa with a theoretical MW of 71.612 kDa and a pI of 8.94. The domains of TLR5M and TLR5S and their homologous genes in other species are shown in Figure 1.

**Figure 2:**
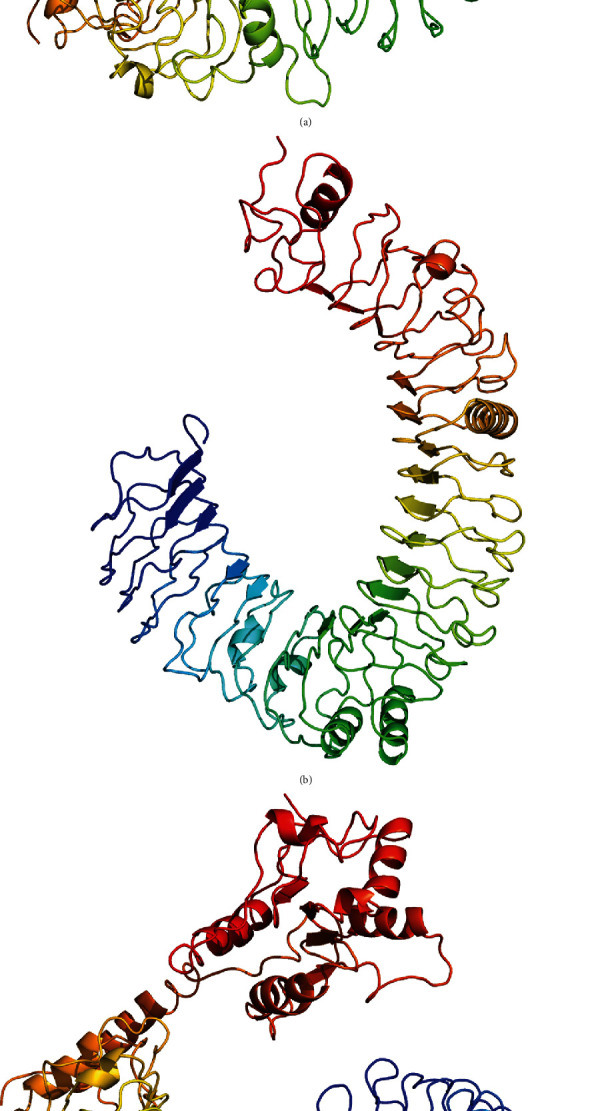


TLR5S lacks TIR and transmembrane domains compare to TLR5M, with an additional LRR_NT and 3 LRRs. The TLR13 gene had a CDS length of 2745 bp and encoded a protein of 914 aa. The calculated MW of the protein was 103.727 kDa, and the pI was 8.20. The tertiary structure of TLR5M, TLR5S, and TLR13 is shown in Figure 2. According to the predicted results, the extracellular structures of the three proteins were very similar, forming a horseshoe shape. The extracellular region of TLR5M contained 18 *β*-sheets, 14 all located in the horseshoe-shaped concave surface, while the 7 *α*-helices were located on the horseshoe-shaped convex surface, and the rest were irregularly curled; the intracellular region was 5 *α*-helix surrounded by 4 parallel *β* fold. TLR5S had only the extracellular region, and its concave surface had 19 *β* folds, and the convex surface has 7 *α*-helices. TLR13 was composed of an intracellular region, a transmembrane domain, and an intracellular region. The extracellular region had 19 *β*-sheets and 7 *α*-helices, and the intracellular region was also surrounded by 6 *α*-helices with 4 *β*-sheets4 (Figure 2(c)).

**Figure 3:**
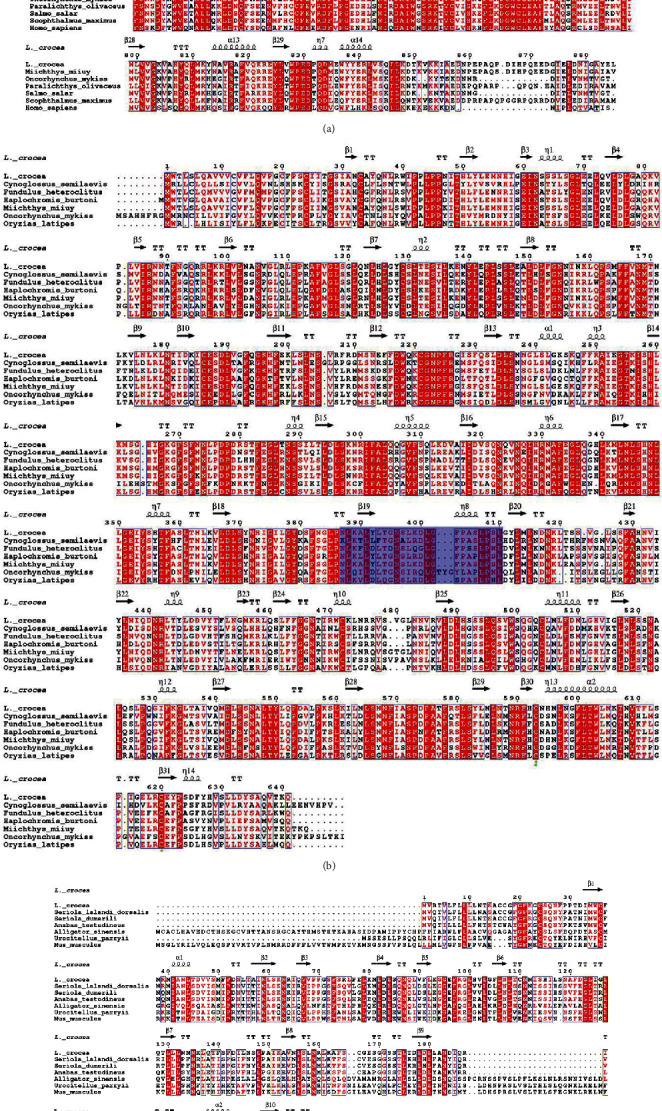


The multisequence alignment of TLR5M and other fish TLRs is shown in Figure 2. The LRR_CT (586-638 aa) of TLR5M is capable of stabilizing the extracellular horseshoe structure, containing a cysteine cluster of CxCx(24)Cx(20)C. Among them, C590 and C616, C592 and C637 could form disulfide bonds, respectively, which is the key to the stability of LRR_CT to maintain the extracellular domain of TLR5M, and these four amino acid residues are conserved in TLR5M of all fish (Figure 3).

### 3.2. Expression of TLR Genes after *V. parahaemolyticus* Challenge (Figure 4)

In this study, in order to detect the expression of TLR5M, qtPCR was used to determine the expression in 12 tissues from healthy *L. crocea*. As shown in Table 2, TLR5M was detectable in all tissues. A relatively high expression level of lc TLR5M (89.3-fold) was observed in the heart, followed by the brain (~36.0-fold) and liver (~8.0-fold). In addition to muscle, the expression of lc TLR5M detected in the spleen was pretty low (1.3-fold); unlike TLR5M, TLR13 had the highest expression in the spleen (182.6-fold), followed by the liver and heart, with the lowest expression level in the brain. Except for the spleen, the expression level of TLR13 in all other tissue was lower than that in muscle. Expression profiles of TLR5M and TLR13 after challenging *V. parahaemolyticus* caused upregulation of the expression level of TLR5M in the spleen, liver, and kidney of *L. crocea*. The expression level of TLR5M in the spleen increased during 0-12 h and reached the highest value at 12 h and then decreased.

However, its peak expression in the liver and kidney appeared at 6 h. In addition, the expression level of TLR5M in the liver increased sharply at 6 h and suddenly decreased after 6 h; in the *L. crocea* treated with *V. parahaemolyticus*, the expression levels of TLR13 in the three immune tissues were upregulated, while comparing with the expression pattern of TLR5M, the fluctuation of TLR13 express was lagging behind, the peaks in the liver and kidney appeared at 12 h, and the peak in the spleen appeared at 24 h; it was apparent that the expression level of TLR13 in the kidney was greatly modulated by *V. parahaemplyticus*.

### 3.3. Phylogenetic Analysis

**Figure 5:**
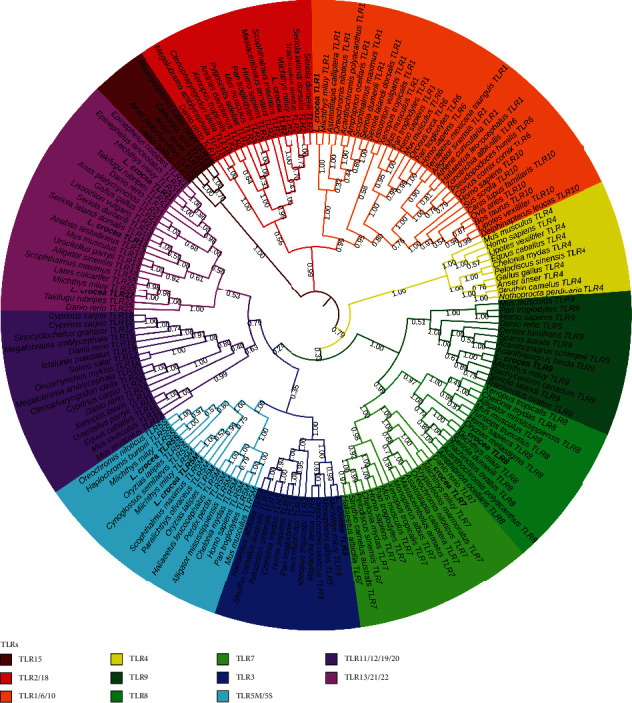
Phylogenetic tree of TLRs based on full-length amino acid sequences. Phylogenetic trees were constructed based on the amino acid sequences of TLRs from fish, birds, and mammals using the neighbor-joining method in MEGA 7. Numbers on the branches represent the percentage of bootstrap values. Node values represent the bootstrap confidence from 10,000 replicates. The GenBank accession numbers of the sequences are listed in Table 1. All TLRs were clustered into 4 groups, in which TLR15 and TLR4 were grouped separately (TLR15 group and TLR4 group), TLR1, 2, 6, 8, and 10 were clustered together (TLR1 group), and the remaining TLRs formed a large group (TLR3, TLR5M/S, TLR7, TLR8, TLR9, TLR11, TLR12, TLR13, TLR19, TLR20, TLR21, and TLR22). The large groups of 13 TLRs could be subdivided into 3 groups: TLR7, 8, and 9 clustered into one group (TLR7 subgroup); TLR3 and TLR5 clustered together (TLR3 subgroup); TLR11, 13, 19, 20, 21, and 22 are clustered into a group (TLR11 subgroup). Vertebrate TLRs were divided into six major TLR families (TLR1, TLR3, TLR4, TLR5, TLR7, and TLR11).

Phylogenetic analysis to elucidate the evolutionary development history of TLR5M, TLR5S, and TLR13, phylogenetic tree analysis on TLRs of *L. crocea*, and other species was performed (Figure 5).

All TLRs were clustered into 4 groups, in which TLR15 and TLR4 were grouped separately (TLR15 group and TLR4 group), TLR1, 2, 6, 8, and 10 were clustered together (TLR1 group), and the remaining TLRs formed a large group (TLR3, TLR5M/S, TLR7, TLR8, TLR9, TLR11, TLR12, TLR13, TLR19, TLR20, TLR21, and TLR22). The large groups of 13 TLRs could be subdivided into 3 groups: TLR7, 8, and 9 clustered into one group (TLR7 subgroup); TLR3 and TLR5 clustered together (TLR3 subgroup); TLR11, 13, 19, 20, 21, and 22 clustered into a group (TLR11 subgroup). Previously, vertebrate TLRs were divided into six major TLR families (TLR1, TLR3, TLR4, TLR5, TLR7, and TLR11) according to evolution. Our evolutionary analysis results were similar to their conclusions, but TLR3 and TLR5 were classified into one family, and the TLR11 family was complemented by TLR19 and TLR20.

### 3.4. Subcellular Localization of TLR5S, TLR5M, and TLR13

**Figure 6:**
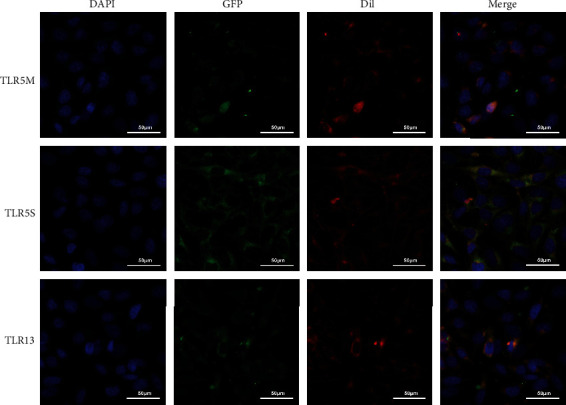
The subcellular localization of TLR5M, TLR5S, and TLR13 in LO2 cells. Panels of the first line were TLR5M, TLR5S, and TLR13 proteins, respectively. The middle panels were the cell nucleus stained with DAPI. Panels of the last line were the merged images of immunofluorescenced protein and DAPI, GFP, and Dil.

The correct localization of a protein in a cell is crucial to understand the function of the protein. Hence, we examined the subcellular localization of TLR5M, TLR5S, and TLR13 (Figure 6). As the results show, TLR5M appeared in the nucleus while TLR5S were cytoplasmic in distribution. TLR13 protein was distributed in the endosome; its proteins were localized in the cytoplasm in a punctuate manner.

## 4. Discussion

### 4.1. Sequence Characteristics and Protein Structure Prediction

The functional domains of TLRs were highly conserved, and the differences were mainly expressed in the N-terminal LRR domain. The LRR domain is the extracellular domain of the TLR and is involved in the recognition and binding of ligands, so its N-terminal differences should have an effect on TLR recognition and binding ligands.

Recent studies demonstrate that piscine contains two types of TLR5, namely, membrane form of TLR5 (TLR5M) and soluble form of TLR5 (TLR5S), both of which perform a crucial role in immunity response [27].

It was obvious that TLR5M had 11 LRRs, 1 LRR_CT, a transmembrane domain, and a TIR domain, which was one LRR less than *Miichthys miiuy* TLR5M and two more LRRs than the human TLR5M. TLR5S had only the extracellular region, and its concave surface had 19 *β* folds, and the convex surface has 7 *α*-helices. TLR5M and TLR5S were also studied in a few fish species, such as *Pelteobagrus vachelli*, *Epinephelus coioides*, *M. miiuy*, *Pangasianodon hypophthalmus*, and *Paralichthys olivaceus* [27–31].

Although the functional domain predicted structure of TLR5M did not have LRR_NT, its N-terminus contained a typical 24Cx(8)C33 cysteine cluster of LRR_NT and was conserved almost exclusively among all fish TLR5M. LRR_NT (19-50) of TLR5Ss also had a Cx(8)C cysteine cluster, which was conserved between humans and fish (Figure 1). LRR_CT (589-640 aa) had only a pair of conserved Cys residues capable of forming disulfide bonds (C593 and C620 for TLR5S). Both fish TLR13 LRR13 (642-691 aa) and mammalian TLR13 LRR_CT had 4 conserved Cys residues (C646, C648, C673, and C690 in fish TLR13; C646, C648, C673, and C691 in mammalian TLR13), too. These cysteine residues form a CxCx(24)Cx(16)C motif (Figure 1). Although TLR13 did not have LRR_NT, it had a conserved Cx(11)C cysteine motif. Hwang et al. experimentally obtained the sequence of the human TLR5M-binding region of flagellin (KLQTLDLRDNALTTIHFIPSI) [32]. The corresponding sequence in TLR5M was NLRGLFLTGNSLLRDLGFPASLPNL, in which fish TLR5M was conserved with motif xLxxLxLTGNSL/IRxLGx(n)AxLPNL/I, while the fish TLR5S also contained similar conserved motifs (blue square markers).

TLRs have a high conservation in the functional domain, and the difference is mainly reflected in the LRR domain at the N-terminal; the difference between TLR5M/S and TLR13 and other TLRs is the number, type, and distribution of LRR at the N-terminal. LRR domain is the extracellular domain of TLR, which is involved in the recognition and binding of ligands, so the difference of the N-terminal of the LRR domain should influence the recognition and binding of TLR. Multiple sequence alignments revealed that the TLR5M/S and TLR13 sequences contained multiple conserved Cys residues that were likely to form disulfide bonds to help maintain the stability of the extracellular structure of TLRs (Figure 2(c)). Previous studies on two types of TLR5 in *Ctenopharyngodon idellus* yielded similar result sequences of TLR5M/S, and TLR13 contain multiple conserved Cys residues, which are likely to form disulfide bonds to help maintain the stability of the extracellular structure of TLRs (Figure 2). Similar results were obtained from previous studies on two forms of TLR5 in grass carp.

The length and functional domain structure of TLR13 is similar to that of *Seriola lalandi dorsalis* TLR13 (sldTLR13), but TLR13 has one LRR less than sldTLR13, while sldTLR13 had one N-terminal LRR-TYP more than TLR13 (Figure 2(c)). LRR number of mice TLR13 was much more than fish TLR13, with 11 LRR and 7 LRR_TYP, but LRR_CT is missing. Comparing all of the various types of TLRs, TLR5Ss had only the LRR domain and TLR13s lacked the transmembrane domain, while other TLRs had TIR, transmembrane domain, and LRRs. TLR5Ss lacked the C-terminal TIR and transmembrane domains, so they were the shortest (around 600 bp). Fish TLR13 has been well studied. For example, Liang et al. studied TLR13 (gTLR13) of the grouper (*E. coioides*), the cDNA of gTLR13 is 3559 bp in length, and the ORF is 2844 bp in length, encoding a protein of 947 amino acid residues (aa). Its domain includes a signal peptide, 13 LRR, a C-terminal LRR, a transmembrane domain, and a TLR structure [33].

Analysis of TLR sequences revealed that all three functional domains (LRRs, transmembrane, and TIR domains) of TLRs, except TLR5S (without transmembrane and intracellular TIR domains) and TLR21 (transmembrane domain deletion), are available (Figure 2). The difference between TLR5M/S and TLR13 of *L. crocea* and other species was in the number, type, and distribution of N-terminal LRRs 41 [34].

### 4.2. Expression of TLR Genes after *V. parahaemolyticus* Challenge

Transcriptional expression of lcTLR5/TLR13 and genes in the TLR signal pathway TLR5 is located on the cell membrane, and it is capable of detecting flagellin and specifically recognizing a relatively conserved constant domain among different bacteria. TLR13 is a TLR that localizes to endosomes and recognizes nucleic acids released by internal microorganisms, including viruses, bacteria, and fungi [35].

Studies have shown that stimulation of *C. irritans* can cause an increase in TLR5M and TLR5S transcription levels in the fins and spleen of *E. coioides* [29]. The expression of *M. miiuy* TLR5S in the liver and kidney increased significantly after infection by *Vibrio harveyi* [28]. Jiang et al. used flagellin and LPS to infect *S. maximus* and found that TLR5M was upregulated in fin, head, kidney, and spleen [35]. In addition, TLR13 in the grouper spleen can be stimulated by the 19-mer S. aureus 23S ribosomal RNA-derived oligoribonucleotide (ORN Sa19) [29]. It has been reported that human TLR5 was mainly expressed in the ovary and was expressed in monocytes, immature dendritic cells, and epithelial cells simultaneously [36]. However, mouse TLR5 was mainly expressed in the liver and lungs [37]. In this study, in order to detect the expression of TLR5M, qPT-PCR was used to determine the expression in 12 tissues from healthy *L. crocea*. As shown in Figure 3, TLR5M was detectable in all tissues. A relatively high expression level of TLR5M was observed in the heart, followed by the brain and liver. In addition to muscle, the expression of TLR5M detected in the spleen was pretty low. Unlike lcTLR5, TLR13 had the highest expression in the spleen, followed by the liver and heart, with the lowest expression level in the brain (Figure 6). Except for the spleen, the expression level of TLR13 in all other tissues was lower than that in muscle.

### 4.3. Phylogenetic Analysis

From the phylogenetic tree, we found that all TLRs were clustered into 4 groups, and the TLR5M/S and TLR13 were clustered in one group, although the genetic distance between them is not so close as that between TLR5M/S and TLR13, which may be the reason for the similar 3D model of TLR5M. All TLRs were clustered into 4 groups, in which TLR15 and TLR4 were grouped separately (TLR15 group and TLR4 group), TLR1, 2, 6, 8, and 10 were clustered together (TLR1 group), and the remaining TLRs form a large group (TLR3, TLR5M/S, TLR7, TLR8, TLR9, TLR11, TLR12, TLR12, TLR19, TLR20, TLR21, and TLR22); the large groups of 13 TLRs could be subdivided into 3 groups: TLR7, 8, and 9 clustered into one group(TLR7 subgroup), TLR3 and TLR5 clustered together (TLR3 subgroup), and TLR11, 13, 19, 20, 21, and 22 clustered into a group (TLR11 subgroup); previously, vertebrate TLRs were divided into six major TLR families (TLR1, TLR3, TLR4, TLR5, TLR7, and TLR11) according to evolution. Our evolutionary analysis results were similar to their conclusions, but TLR3 and TLR 5 were classified into one family, and the TLR 11 family was complemented by TLR19 and TLR20.

### 4.4. Subcellular Localization of TLR5S, TLR5M, and TLR13

As per the previous studies, there two types of TLR5 of fish, namely, membrane form of TLR5 (TLR5M) and soluble form of TLR5 (TLR5S) [38]. Interestingly, TLR5M appeared in the nucleus while TLR5S were cytoplasmic in distribution in *L. crocea*, which was different from other fish; TLR13 localized in endosomes, which basically recognize nucleic acids released by intracellular microorganisms, including viruses, bacteria, and fungi. In this study, TLR13 of *L. crocea* protein was distributed in endosomes; its proteins were localized in the cytoplasm in a punctuate manner.

## Figures and Tables

**Figure 4:**
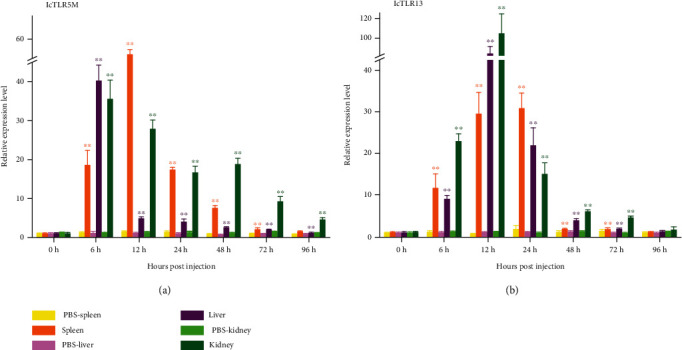
Gene expression profiles of TLR5M and TLR13. Their expression levels were normalized to that of *β*-actin and compared to their expression levels in the muscle. The results are presented as the mean ± SE of fold changes, and ^∗^ and ^∗∗^ indicate statistical significance at *p* < 0.05; ^∗∗^*p* < 0.01.

**Table 1:** The GenBank accession numbers of TLR amino acid sequences.

Species	Protein	Accession no.	Species	Protein	Accession no.
*L. crocea*	TLR1	AHB51065.1	*Miichthys miiuy*	TLR1	AKJ66261.1
	TLR2	AHB51066.1		TLR2	AFG21856.1
	TLR3	KKF15845.1		TLR3	ALJ55565.1
	TLR7	AGO28200.1		TLR5S	ALJ55567.1
	TLR8	XP_010741343.3		TLR5M	ALJ55566.1
	TLR9	ACF60624.1		TLR8	ALJ55569.1
	TLR21	AOZ21302.1		TLR9	ALJ55570.1
	TLR22	ADK77870.1		TLR21	ALJ55573.1
*Lates calcarifer*	TLR22	AOV82293.1		TLR22	ALJ55574.1
*Acanthochromis polyacanthus*	TLR1	XP_022069587.1	*Amphiprion ocellaris*	TLR1	XP_023131471.1
*Cathartes aura*	TLR15	KFP51840.1		TLR8	XP_023148269.1
*Scophthalmus maximus*	TLR1	ANS71060.1	*Alligator sinensis*	TLR1	XP_006029305.1
	TLR22	AIC75880.1		TLR13	XP_006015139.1
	TLR2	ANV20861.1	*Andrias davidianus*	TLR2	AHB18364.1
	TLR5M	AMQ35501.1	*Anabas testudineus*	TLR13	XP_026206843.1
*Seriola lalandi dorsalis*	TLR1	XP_023251851.1	*Seriola dumerili*	TLR1	XP_022607278.1
	TLR2	XP_023279904.1		TLR2	XP_022625698.1
	TLR9	ALI16362.2		TLR8	XP_022597011.1
	TLR13	XP_023266978.1		TLR13	XP_022598134.1
*Oreochromis niloticus*	TLR1	XP_013126527.2	*Lissotriton vulgaris*	TLR1	AIZ71264.1
*Xenopus laevis*	TLR12	XP_018082190.1		TLR21	AIZ72110.1
	TLR3	XP_003449776.2	*Pelodiscus sinensis*	TLR3	XP_006128521.1
	TLR5S	XP_019201018.2		TLR4	NP_001273862.1
	TLR7	XP_019208379.1		TLR7	XP_014428945.1
*Corvus cornix cornix*	TLR6	XP_010400098.3	*Haliaeetus albicilla*	TLR3	XP_009914242.1
*Phoenicopterus ruber ruber*	TLR15	KFQ89147.1		TLR7	KFP93553.1
*Ctenopharyngodon idella*	TLR18	AIB55030.1	*Struthio camelus*	TLR4	AKP92940.1
	TLR20	AHN49762.1	*Equus caballus*	TLR4	NP_001093239.2
Epinephelus lanceolatus	TLR21	AJW66342.1		TLR11	XP_001502488.1
*Mastacembelus armatus*	TLR2	XP_026178049.1	*Haliaeetus leucocephalus*	TLR2	AVY54488.1
	TLR3	XP_020470010.1		TLR5M	XP_010575989.1
	TLR7	XP_026150467.1	*Urocitellus parryii*	TLR11	XP_026267776.1
*Oryzias melastigma*	TLR8	XP_024141916.1		TLR13	XP_026261358.1
*Haplochromis burtoni*	TLR5S	XP_005931636.1	*Gallus gallus*	TLR4	ACR26292.1
*Epinephelus coioides*	TLR21	ADM34974.2		TLR15	NP_001032924.1
*Alligator mississippiensis*	TLR5M	XP_019338679.1		TLR21	NP_001025729.1
	TLR8	XP_014463251.1	*Anser anser*	TLR4	AEC32857.1
*Pseudopodoces humilis*	TLR6	XP_005518093.1		TLR15	AFK25800.1
*Danio rerio*	TLR7	XP_021334735.1	*Oryzias latipes*	TLR5S	XP_004083935.2
	TLR9	NP_001124066.1		TLR5M	XP_004083724.1
	TLR18	XP_021322343.1	*Dentex tumifrons*	TLR9	ABY79218.1
	TLR19	NP_001352353.1	*Zonotrichia albicollis*	TLR6	XP_005490558.1
	TLR20	NP_001170914.2	*Orcinus orca*	TLR6	XP_012387211.1
	TLR21	NP_001186264.1	*Sparus aurata*	TLR9	AAW81697.1
	TLR22	NP_001122147.2	*Tursiops truncatus*	TLR8	XP_019784091.1
*Acanthopagrus schlegelii*	TLR9	ABY79216.1	*Delphinapterus leucas*	TLR10	XP_022421795.1
*Rachycentron canadum*	TLR9	AGD79973.2	*Sinocyclocheilus grahami*	TLR12	XP_016140610.1
*Cyprinus carpio*	TLR12	XP_018974718.1	*Megalobrama amblycephala*	TLR18	APT35507.1
	TLR19	BAU98390.1		TLR19	APT35508.1
	TLR20	AHH85805.1		TLR20	APT35509.1
*Ictalurus punctatus*	TLR19	AEI59675.1	*Terrapene mexicana triunguis*	TLR1	XP_024077328.2
*Astatotilapia calliptera*	TLR1	XP_026048804.1	*Takifugu rubripes*	TLR21	NP_001027751.1
*Astatotilapia calliptera*	TLR7	XP_025999893.1	*Acanthopagrus berda*	TLR9	ABY79215.1
	TLR1	XP_026048804.1	*Takifugu rubripes*	TLR21	NP_001027751.1
*Struthio camelus australis*	TLR7	KFV85786.1	*Acanthopagrus berda*	TLR9	ABY79215.1
*Paralichthys olivaceus*	TLR5M	AEN71824.1	*Struthio camelus australis*	TLR3	KFV82215.1

**Table 2:** Primers used for gene expression analysis by qRT-PCR assay.

Primer	Sequence (5′→3′)	Primer	Sequence (5′→3′)
TLR5M-F	CCTTATCATCACGGTTGT	TLR13-F	AAACTAATTCTTTACCGGACAG
TLR5M-R	ACAGGAGGCATCGGTTTT	TLR13-R	ATGTCCAAAGCACGCAAT

## Data Availability

The data used to support the findings of this study are included within the article: Wu, C., Zhang, D., Kan, M. et al. The draft genome of the large yellow croaker reveals well-developed innate immunity: Nat Commun 5, 5227 (2014). 10.1038/ncomms6227, and the BioProject accession number is PRJNA237858.
